# Perception of couples’ on multipurpose prevention technology attribute choice: the case of MTN 045

**DOI:** 10.1186/s12889-024-19390-0

**Published:** 2024-07-15

**Authors:** Adlight Dandadzi, Alinda M. Young, Petina Musara, Mary Kate Shapley-Quinn, Doreen Kemigisha, Prisca Mutero, Nyaradzo M. Mgodi, Juliane Etima, Alexandra A. Minnis

**Affiliations:** 1 University of Zimbabwe-Clinical Trials Research Centre, Harare, Zimbabwe; 2 Women’s Global Health Imperative, RTI International, Berkeley, CA USA; 3 Department of Maternal and Child Health, University of North Carolina, Chapel Hill, NC USA; 4 Makerere University-Johns Hopkins Research Collaboration, Kampala, Uganda

**Keywords:** Multipurpose prevention technology, HIV, Pregnancy, Eastern and Southern Africa, Couples

## Abstract

**Background:**

Multipurpose prevention technologies (MPTs) are products capable of simultaneously addressing multiple sexual and reproductive health needs such as unwanted pregnancy, STIs including HIV-1, and other reproductive tract infections. MPTs are urgently needed to address the double burden of unplanned pregnancy and HIV. While condoms are currently the only accessible MPTs, they are not solely under a woman’s control, and female condoms face limitations due to poor acceptability and high cost.

**Methods:**

We conducted a sub-analysis of qualitative data from 39 couples participating in the MTN 045 study to examine the perception of couples on choice and acceptability of a “2 in 1” MPT that combines HIV and pregnancy prevention.

**Results:**

Couples recognized the benefits of MPTs for HIV and pregnancy prevention but perceptions tied to each indication and a novel prevention technology tool raised important concerns relevant to use of future MPTs. In the study, participants’ perceptions of MPT use were influenced by pregnancy planning. When the timing was less critical, they prioritized HIV prevention. Misinformation about family planning methods, including MPTs, affected decision-making with potential to hinder uptake of future MPTs. Concerns about side effects, such as weight gain and hormonal imbalances, influenced willingness to use MPTs.

**Conclusion:**

Addressing the myths and misconceptions surrounding the use of contraceptives is crucial in promoting their acceptance and ultimate use. Strategies for addressing the drawbacks women might experience while using a particular product should be in place as new MPTs progress through the development pipeline and approach roll-out.

## Introduction

Globally, more than 37.7 million people live with HIV, and an additional 2 million people become newly infected each year [1]. The Eastern and Southern Africa region is disproportionately affected by HIV, with over 70% of all people living with HIV globally residing there [2]. Furthermore, women of child-bearing age in this region bear a heavy burden of unintended pregnancies and sexually transmitted infections (STIs), including HIV [3]. The acceptability of HIV prevention methods in this region may greatly contribute to the success and potentially have a significant impact on the global burden of the disease.

Daily oral pre-exposure prophylaxis (PrEP) is an effective HIV prevention method for heterosexual women and men who have sex with men and has been implemented through national programs and demonstration projects in the region since 2016 [4–6]. Additionally, the monthly dapivirine vaginal ring has recently proven efficacious in preventing HIV acquisition in the region and has received regulatory approval in several African countries, including Uganda, Zimbabwe, Zambia, Lesotho, South Africa, and Kenya [7–9]. In 2022, the World Health Organization issued a guideline recommending long-acting injectable cabotegravir (CAB-LA) as an HIV prevention option, with Zimbabwe being the first African country to approve CAB-LA for use [10]. While these interventions are effective for HIV prevention, they do not address the unmet need for contraception. Unintended pregnancies pose significant health and economic burdens in low and middle-income countries. Globally, nearly half of all pregnancies (over 100 million per year) are unintended [11]. Alarmingly, more than half of these unintended pregnancies occur due to inconsistent and incorrect contraceptive use, even when women are utilizing contraceptives [12].

Preventing HIV and unintended pregnancies remains a critical priority for women, particularly in Sub-Saharan Africa. While the use of condoms, the sole existing multipurpose method, offers reliable protection against STIs and unintended pregnancy, the uptake and consistent use of condoms is low in the region [13, 14]. To address these challenges, multipurpose prevention technologies (MPTs), also known as “combination” or “dual” technologies, have been developed to simultaneously address multiple sexual and reproductive health needs, including unintended pregnancy and STIs, including HIV [11]. Results of previous studies have shown that women may find a product that prevents HIV and pregnancy more acceptable and likely to be used [15]. However, the influence of existing perceptions regarding HIV and pregnancy prevention methods on the acceptability and use of MPTs in Eastern and Southern Africa remains poorly understood.

To bridge this knowledge gap, it is crucial to investigate how perceptions regarding HIV and pregnancy prevention methods impact women’s choices regarding their sexual and reproductive health. Therefore, the Microbicide Trials Network (MTN)-045 main study aimed to evaluate couples’ preferences for MPT product attributes and understand the decision-making process within a relationship context. In this analysis, we specifically examine how perceptions influence preferences for future multipurpose prevention options in development and affect women and men’s decision-making. By contributing to the limited evidence base on the perceptions influencing multipurpose prevention product preferences, we aim to contribute to the reduction of HIV infections and unintended pregnancies in the region.

## Methods

### Study design and procedures

MTN-045 was a cross-sectional study investigating the factors influencing the acceptability of potential MPT product options in development by assessing couples’ preferences regarding different forms of drug delivery. The different forms of drug delivery assessed were the vaginal ring, vaginal insert, vaginal film and oral pills. The study was conducted in Kampala, Uganda and Chitungwiza, Zimbabwe between January and November 2020. A total of 400 heterosexual couples, comprised of 200 couples in Uganda and 200 couples in Zimbabwe, were enrolled in the study. Further details on the study design and setting have been previously reported elsewhere [13, 19].

A total of 39 couples were purposively selected for qualitative in-depth interviews (IDIs) after completing individual and joint discrete choice experiments (DCEs). Each site research team invited up to 20 couples to participate in IDIs, conducted either with both partners together (*N* = 19) or separately (*N* = 20), for a total of 59 interviews. For the separate interviews, there were 20 couples who participated which equalled 40 individual interviews. The aim was to have an equal number of interviews conducted with couples together or separately, representing various decision-making dynamics and relationship lengths. The qualitative interviews took place as a separate visit within one month of the main study visit. A semi-structured interview guide was used, focusing on couple dynamics and their effects on HIV prevention and contraceptive decisions. The IDIs focused on communication and decision-making within the relationship, encompassing sexual and reproductive health decisions as well as general decisions. The interviews also covered descriptions of HIV and pregnancy prevention practices, challenges and facilitators encountered with contraceptive and HIV prevention methods, and factors influencing use of potential future MPTs (such as situational, relationship, social/cultural/economic, and sex and menstruation-related factors). Trained interviewers ensured neutrality and encouraged detailed responses. The interviews were conducted in English and/or Luganda (in Uganda) and Shona (in Zimbabwe). The interviews lasted approximately 60 min, were digitally recorded, and underwent transcription and translation processes. To ensure the trustworthiness of the data, an interviewer, different from the one who would have conducted the interview, simultaneously transcribed and translated the in-depth interview. The interviewer, who conducted the interview, reviewed the transcript in its entirety against the audio to ensure it reflected the content of the interview. The Qualitative Coordinator conducted in-depth reviews of the first 3 transcripts from each interviewer, to provide feedback on moderating and interviewing techniques (e.g. adequacy of probing, appropriate linking of topics, fidelity to the guide, etc.). Following this determination, quality checks included listening to at least three 5-minute spots in the audio file as compared to the transcript. The text of each transcript was reviewed in its entirety even if the entire audio file was not reviewed, and the de-identified transcripts were stored securely. More detail regarding the qualitative sample has been published elsewhere [16].

### Data analysis

After each interview, interviewers recorded key themes using a debriefing report template. These reports were reviewed by the protocol team to assess interview quality, identify preliminary themes, and determine areas for further investigation. The preliminary codebook was derived from these reports and the questions in the interview guide. Two randomly selected transcripts were reviewed by six team members to add additional codes. The codebook was then reviewed by the study team to address gaps and redundancies, and two co-authors revised it to create the final version. Four coders independently applied the codes to 59 transcripts (19 joint interviews and 40 individual interviews). To ensure consistency, the study team coded 8–10 transcripts per week, holding weekly meetings to resolve discrepancies and reach consensus.

We conducted a thematic content analysis of the data focusing on codes that included “gender norms”, “HIV and Communication”, “influencers”, “stories and stigma”, and “fertility”. The lead author, supported by a qualitative analyst, reviewed the summary code reports and read the transcripts one by one several times, paying attention to shared processes to determine meaningful components in the narratives that provided an understanding of the perceptions and their effects on an MPT product attribute choice. We focused on the following question when rereading the transcripts: How do perceptions influence product attribute choice among the married and unmarried participants? Upon obtaining a sense of immersion in the data, the authors met to discuss initial findings. The findings were reported by describing the responses of the participants using the themes identified. Our reporting is aligned with the consolidated criteria for reporting qualitative research (COREQ) [17].

### Ethical considerations

The study was approved by the following Institutional Review Boards and Ethics Committees: Medical Research Council of Zimbabwe; Joint Research Ethics Committee for the University of Zimbabwe, Faculty of Medicine and Health Sciences and Parirenyatwa Group of Hospitals; Research Council of Zimbabwe; Chitungwiza City Health Department; Joint Clinical Research Centre Research Ethics Committee; Uganda National Council for Science and Technology; Johns Hopkins School of Medicine Institutional Review Board; Advarra Institutional Review Board. Participants provided written informed consent and confirmed their consent verbally before the interviews.

## Results

A total of 39 couples (19 joint interviews and 40 individual interviews) participated in the IDIs. The average age of females was 25.7 years, ranging from 18 to 38 years, while males had an average of 30 years, ranging from 19 to 45 years. 64% of the females had completed secondary school compared to 74% of the males. The most used method for contraceptive was oral pills with 33% of the females using it whilst the least used method was intrauterine device (IUD) with 3%; 18% did not use any contraceptive method. 26% of the females used male condoms, while 13% used implants and 15% used injectables. Natural contraceptive methods which included rhythm, fertility awareness, and calendar were used by 8% of the females. Other contraceptive methods like female condoms, emergency contraception, female sterilization, and withdrawal, were utilized by 10% of the females. There were 2 (5%) females who were pregnant during the study period whilst 8 (21%) were breastfeeding. The average years of partnership relationship was 5.4 years with the longest being in existence for 21 years and the least just 0.7 years. In general, males were older than females in a partnership, with a mean age difference of 4.0 years. Of the 39 couples, 46% were married, 82% were either married or cohabitating and 62% had children together. Family planning decisions were jointly made by 74% of the couples and 36% of the couples reported using a method for HIV prevention. The demographic characteristics of participants, are presented in Table 1.

**Table 1:** Sociodemographic characteristics of couples: qualitative subsample in MTN-045

	*N*	(%)
Total couples	39	(100)
Location		
Kampala, Uganda	20	(51)
Chitungwiza, Zimbabwe	19	(49)
***Female partner***		
Age, years - *mean*,* median (range)*	25.7, 25	(18–38)
Completed secondary school	25	(64)
Parous	29	(74)
*Current contraceptive method(s)*		
Oral pills	13	(33)
Injectable	6	(15)
Implant	5	(13)
IUD	1	(3)
Male condoms	10	(26)
Natural methods^1^	3	(8)
Other^2^	4	(10)
None	7	(18)
NA – currently pregnant	2	(5)
Currently breastfeeding	8	(21)
***Male partner***		
Age, years - *mean*,* median (range)*	30.0, 30	(19–45)
Completed secondary school	29	(74)
***Partnership characteristics***		
Relationship length, years - *mean*,* median (range)*	5.4, 3	(0.7–21)
Age difference, years^3^ - *mean*,* median (range)*	4.0, 3	(-7-14)
Married	18	(46)
Married or cohabitating	32	(82)
Have children together	24	(62)
Family planning decisions made jointly	29	(74)
Currently using a method for HIV prevention^4^	14	(36)

^1^ Methods included rhythm, fertility awareness, calendar

^2^ Female condoms, emergency contraception, female sterilization, withdrawal

^3^ Male partner’s age minus female partner’s age

^4^ Either partner reported using method currently

### Overview of qualitative themes

The main topical areas in which perceptions related to pregnancy and HIV emerged as influential to MPT choices and decision-making included: general reactions to an MPT, delaying pregnancy, community perceptions and misconceptions, concerns about an MPT, and influence of religion in use of HIV and family planning methods. These topic areas and sub themes are explored below.

### General reactions to an MPT

#### Preference influences

Couples liked the idea of a dual-purpose product citing that using the MPT is like “hitting two birds with one stone” because it would allow women to simultaneously prevent HIV and pregnancy. Participants also highlighted that an additional advantage of using an MPT was that it minimizes the chances of one forgetting to take the product every day, as illustrated by a participant below.

*…This one helps a lot and it is the best because it has two advantages; it prevents me from HIV infection and pregnancy. She might be using pills for two purposes but she forgets to take that day and she has sex then she can conceive but if she uses this two in one method then she has protection against the two and that is it. That method is good… that is the reason that might influence us; that one stone hits two birds [2178*,* Female*,* Uganda]*.

Additionally, participants noted that MPTs provided an opportunity to ensure the woman was protected against HIV infection, especially among serodiscordant couples or in cases where the woman believes her husband or partner is being unfaithful. One male partner from Uganda, illustrates this point below.

*If I am infected from another person at least my wife has that two in one method then she cannot be infected with that virus. The advantage is that they will neither be infected with HIV or conceive [21045*,* Male*,* Uganda]*.

Though women acknowledged the benefits of the MPT, they expressed that it was up to each person or couple to decide whether or not to use it. Some were concerned about an MPT’s limitations and indicated that they would not be interested in using it because it would not cater to their different needs at different times in their lifetime. Participants expressed that the MPT would not be the best option for women who would want to prevent HIV but not pregnancy. Some participants also believed that because it is a multi-purpose product, women would need to switch to a single HIV product if they decided they wanted to become pregnant and to remain HIV free because they would not be able to simply remove the family planning component of the MPT. This would make them more vulnerable to HIV during the anticipated waiting period between discontinuing the MPT and initiating the HIV prevention only product.

### Delaying pregnancy

#### Motivation for using an MPT

Most participants expressed that timing of pregnancy would determine whether couples would choose to use the MPT or not. For married couples, the MPT was seen as a way to assist the couple in spacing children and giving them the freedom to determine when they were ready to add another member to their family. Married couples believed this product would ensure they are financially stable and the children they already have are healthy before adding on another child. For unmarried couples, particularly those with other commitments such as school, the MPT was viewed as a great option that would limit unintended or unwanted pregnancies while at the same time ensuring the woman was protected against HIV. Thus, participants believed men, especially those unprepared to be fathers, would be supportive of their female partners using these products. Furthermore, participants also believed that this product could be used secretly by women in cases where men wanted to keep fathering children or were against the use of contraceptives or HIV prevention methods, as illustrated below.

*They [men] care a little just to provide some support but as a woman you have to program how to space your children. If we get that product (MPT)*,* the woman can program secretly and know that even if the man wants a child*,* but the one I have with you doesn’t have basics*,* no education but you are putting pressure on me to give birth to another child*,* you can use your method secretly until you see the situation improving and decide to get pregnant. This does not happen with the family planning methods which are in place because these are easily noticeable… [2180*,* Male*,* Uganda]*.

Other participants mentioned that some men would not be prepared to bear the responsibility of childbearing; hence, they would support their female partners to use an MPT product. One of the male partners in a joint interview said:

*There are men who fear responsibility*,* they do not want to give birth to children or they might have older children…. He might want to use that product but at the same time it protects from HIV. Some men do not like responsibilities*,* women giving birth to their babies and that might make them want to use the product [22753*,* Male*,* Uganda]*.

### Community perceptions and misconceptions

#### Use of family planning methods myths and misconceptions

Myths and misconceptions surrounding the use of contraception among women came out prominently in the interviews across both sites. Some participants spoke of rumors such as the contraceptive implant disappearing in the body or the pills causing stomach cancer. Other participants mentioned hearing myths about how using contraceptives causes one’s eggs to get burnt thereby delaying or impairing their fertility. Therefore, participants were worried about using a MPT for family planning and they also thought that the community would be worried about it. Participants mentioned hearing stories of local women who used family planning methods but failed to have children after discontinuing. A couple from Uganda voice this concern in the passage below:

**M**:…*we have some of the family planning methods which one can use for some time and when she needs to have another child then the woman may take so long to conceive or she may completely not be able to have another child*…

**F**: *She used it but has looked for a child and totally failed so this may cause fear.*

**M**: *But if it is good then nothing can make us fail to use that method.*

***F***: *If it does not have those side effects like if you want to conceive and you totally fail and when you go to the health facilities you are told that you will not conceive again*,* the eggs in the ovary were all burnt. When people learn about this then they will fear using it but without such side effects then they will use it [2178*,* Couple*,* Uganda]*.

Additionally, condoms, the only current MPT product, were reported by participants to be associated with commercial sex workers and thus often disregarded in relationships or marriages. One of the couples in a joint interview had this to say regarding the use of condoms:

**F**: *You will be afraid to be asked ‘aah condoms are for commercial sex workers?*

**M**: *Why do you need them?*

**F**: *So, if I ask for protection my boy [partner] will say that I’m a what-.*

**M**: *Commercial sex worker.  [4110, Couple, Zimbabwe]*

Of note is that condoms as the only MPTs are insufficient as users of both condoms may encounter some common challenges (such as the belief that using a condom reduces sexual pleasure compared to not using one, the need to negotiate with partners, stigma, and a lack of knowledge or experience with condoms).

Moreover, a few participants were worried that “newness” of this product would lead some individuals to believe manufacturers had malicious intentions. The quotes below illustrates this point regarding “newness” of the MPT:

*… The other concern could be since it is new*,*“how many people have used it so far*,* do you want to kill us?” they want us to stop having many children*,* cause infertility*,* they want to kill us since it is a new method [22423*,* Female*,* Uganda].*

**F**: *Since it is something that you might not have used, that you are not used to, and you have never used before, you will be scared to use it, to say what if it affects me….*

**M**:*…as something that would have been introduced, and you are not used to using it, it can take time for us to get used to it… It is something that we don’t know whether it really protects or not, because it is something that we haven’t used yet. We have the things that we have been used to.*

*[4102*,* Couple*,* Zimbabwe]*

At the same time, sharing of positive experiences with family planning use was viewed to favorably influence others in the community and was suggested as a potential avenue for future MPT products:

*Men whose wives don’t complain encourage their fellow men to allow their wives to use family planning. A mother can influence and encourage her daughter to use it out of her good experience. So*,* it is people’s stories which either encourage or discourage people to use family planning. My wife is just 30 years; she shouldn’t fear family planning [22735*,* Male*,* Uganda]*.

### Concerns about an MPT

#### Side effects

The topic of side effects was heavily debated by couples, but mainly in relation to beliefs and experiences with actual family planning methods. Participants discussed how some women who use family planning methods experience adverse effects including excessive bleeding, decreased sexual desires, weight gain, excruciating cramps, and nausea that often lead to discontinuation of the method and sometimes revert to using traditional methods. Some participants also highlighted how using contraceptives incorrectly can have unfavorable side effects. For instance, some participants mentioned that because women frequently communicate their experiences with contraceptives, if other women hear about the unfavorable side effects, they are typically discouraged from using the same contraceptive or contraception in general. As a result, participants were worried that the same problem would prevent women from taking a MPT product. Additionally, participants were concerned that the side effects from a MPT product might be more severe than they would be with a single prevention strategy, such as oral PrEP or implants. Furthermore, a few male participants mentioned information sharing among male friends and described that some men often forbid their wives or girlfriends from using family planning methods as a result of bad experiences men have recounted regarding the use of the methods by their partners. In the case of one couple, the man claimed that males dislike contraceptive use because the side effects (e.g., excessive bleeding or decreased sexual desire) experienced by the woman as a result of product use sometimes makes it difficult for them to satisfy their sexual needs. Accounts, like those below, suggested that although men do favor family planning, they dislike its adverse effects.

**F**: *The other issue is that most men do not like those family planning methods that we use because of side effects like irregular bleeding so they don’t like them and that’s why in most cases the men refuse the women to use them because you are bleeding and he cannot fulfil his need.*

***M***: *And there is a way they change the woman…there is a way they change the woman… The woman’s sex libido is low and sometimes she doesn’t completely want sex because she has no feelings. [2178*,* Couple*,* Uganda]*.

*…Haa*,* the other reasons that might make us not use this product is that the wife might use the product and have side effects. It might affect her menstrual cycle. It can also … Like during sex*,* it can make her produce a lot of water [vaginal discharge]. It can cause too much dryness. [4102*,* Male*,* Zimbabwe]*.

#### Concerns that future MPTs will increase infidelity and relationship conflicts

While the MPT product was mentioned above to provide protection against HIV and unintended pregnancies, some participants were also concerned that it would lead to promiscuity among women and thus increase infidelity in marriages and relationships. One male participant illustrated this point and said that people have been faithful to each other because they feared contracting HIV or unwanted pregnancies; however, this will no longer be of concern with the use of a MPT product, especially among MPT product users. Male partner concerns about MPTs facilitating infidelity, however, were also tempered by recognition that MPTs could be beneficial to their marriages and relationships because their female partners will be protected against HIV and pregnancy in their absence:

*… the problem of adultery will increase because it will be the woman to commit adultery more than the man because she knows that she cannot get pregnant*,* she cannot get HIV …on the side of men*,* they say it is very good in case they travel out of the country for work*,* they will not find their wives with HIV or pregnancy when they return home. [22195*,* Male*,* Uganda]*.

Some participants were also concerned that the use of a MPT product would potentially lead to relationship conflicts. These participants believed that some women would have challenges convincing their male partners to use a MPT because male partners would attribute the use of the product to mistrust; believing that either the woman had other sexual partners or that she didn’t trust him. Some male participants also spoke of women’s desire to use a MPT product resulting in dissolution of marriages or relationships because it was perceived that male partners are the decisionmakers on when to use or not use pregnancy or HIV prevention methods. The quote below by one man illustrate this point:

*There are other people who [may] say*,* no if I let my wife use this thing that is 2 in 1 that prevents HIV infection and pregnancy*,* she will be found looking for other men from outside as she is confident she is protected from the infection. And then another one can say if I allow my wife to use it she no longer trusts me. She is saying maybe I’m looking for the infection [4103*,* Male*,* Zimbabwe]*.

Moreover, protection against infidelity was not only attributed to the men. Some participants also spoke of women being protected when they themselves had multiple sexual partners. One participant pointed out that:

*It’s not about men only some women too have other sexual relationships so this drug may help her in prevention against HIV [22423*,* Female*,* Uganda]*.

### Religion

#### Influence of religion in use of HIV and family planning methods

Couples from both sites discussed the role played by religion especially in discouraging use of family planning methods. They highlighted the potential for religion to influence whether individuals used the MPT. Participants believed that while everyone would want to prevent HIV, some individuals would deter away from using a MPT product because it contained a contraceptive and prevention of pregnancy is viewed by some religious individuals as “killing” of an unborn fetus. Therefore, couples or individuals would rather seek HIV treatment in order to stay true to their religious beliefs than use a MPT product. The narration below demonstrates the power of religious beliefs in the context of healthcare seeking behaviour:

**M**: *There are some who don’t want their wife to get even a Jadelle… There are also people from the apostolic sect [churches] who don’t allow their wives even when giving birth…The wife doesn’t give birth at the hospital…They only allow their wives to give birth at church.*

**F**:… *my mother’s whole family belong to the Maran burg [pseudonym] church so my mother and all her siblings were born at home. Even now even if they get sick, all of them at their rural home, no one goes to the clinic.*

**M**: *They don’t even prevent [pregnancy]. There is no discussion, it’s just bearing children non-stop.*

*[4110*,* Couple*,* Zimbabwe]*

## Discussion

The study revealed that couples expressed positive attitudes towards a MPT product, recognizing its benefits in simultaneously preventing HIV and pregnancy. These results align with findings in the main study where 91% of the couples preferred MPTs over separate HIV prevention and family planning products [18]. Importantly, the study found that couples contributed equally to conversations around 2-in-1 product attributes and preferences. Participants perceived an MPT product as advantageous, particularly in providing protection against HIV for serodiscordant couples and in cases of suspected infidelity. However, concerns were raised regarding the limitations of an MPT product and the potential need to switch to alternative HIV prevention methods to allow for childbearing. The timing of pregnancy emerged as a crucial factor in the decisions regarding MPT interest and anticipated use of a future MPT, with married couples viewing it as a means of planning and spacing children, while unmarried couples saw it as a way to prevent unintended pregnancies. The study also identified various barriers to MPT product acceptance, including misconceptions about contraception, concerns about side effects, and religious beliefs. Relationship conflicts, worries about increased promiscuity, and community perceptions were also noted as potential challenges.

Couples’ positive attitudes towards a MPT product and their recognition of its benefits in preventing both HIV and pregnancy simultaneously highlight the potential of MPT products to address multiple health concerns conveniently. This underscores the importance of developing and promoting integrated prevention strategies. Strategies for addressing the drawbacks women might experience while using a particular product should be in place as new MPTs progress through the development pipeline and approach roll-out [19, 20]. Instead of solely emphasizing the potential benefits that new MPT products would provide to women, these strategies should offer proactive solutions for barriers to product use at the community, facility, partnership, and individual levels [21].

The concerns surrounding the limitations of a MPT, particularly for women who want to prevent HIV but not pregnancy, raise important considerations for policymakers and healthcare providers. Results from a study done in South Africa suggested that a universal HIV prevention strategy will not be an effective or efficient use of resources [22]. This is in line with recent trends in HIV and contraceptive research, where it has become clear that a range of alternatives can maximize uptake and adherence. These findings emphasise the fact that different groups have unique preferences and requirements, justifying the need for choice that supports more individualized prevention programmes [21]. Addressing these concerns and exploring alternative prevention methods is crucial to ensuring comprehensive and tailored HIV prevention options for individuals and couples. It is important to note that perceived disadvantages associated with a MPT may have tangible implications for interest and uptake of a new product, as well as for the available alternative choices, particularly for providers and institutions offering new MPTs to women. Therefore, providers will play a crucial role in assisting each client in determining whether the drawbacks of a MPT will act as a barrier to use, with the understanding that not all drawbacks will have equal salience across potential MPT users [23].

The influence of sociocultural factors, such as misconceptions about contraception and religious beliefs, should be taken into account when designing and implementing MPT programs. Religious beliefs mirror the values and attitudes of communities and individuals, influencing their behavior and practices. Tailored educational initiatives and community engagement are necessary to address misconceptions, dispel myths, and respect individuals’ religious beliefs while promoting the benefits and safety of MPT products [23–25]. A study conducted in Malawi, South Africa, Uganda and Zimbabwe demonstrated the critical role of religious leaders in addressing misconceptions about contraception and religious beliefs in HIV prevention interventions. Participants in this study and others agreed that researchers and religious leaders could collaborate to promote HIV prevention products; however, further research is needed to determine the most effective strategy in involving and building partnerships with religious leaders that lead to them playing a role in promoting use of prevention products [26].

The significance of relationship dynamics and the timing of pregnancy in the decision to use the MPT product highlight the need for comprehensive and client-centered approaches. Our findings complement the results of previous studies, where the topic of power dynamics between men and women and their effect on decisions regarding sex and covert HIV prevention product use were discussed by the participants [22]. However, the desire of some men to participate in shared decision-making regarding their partners’ health issues emerged strongly. The male partners voiced their concerns about their female partners participating in research and utilising products without their knowledge, supporting the accumulating evidence of men influencing the uptake of female-initiated methods [27]. Therefore, healthcare providers should engage both partners in discussions and decision-making processes, considering individual preferences, relationship dynamics, and the specific needs and goals of couples enhance acceptance and adherence to MPT products.

## Limitations

Our study has several limitations to acknowledge. First we did not specifically explore perceptions on MPT product choices, as it was not the primary focus of the study. This limited our ability to delve deeply into this aspect. Additionally, our participants provided their views based on hypothetical scenarios, lacking the real-world usage experiences, which might have yielded different results. However, the role of beliefs and practices influencing attitudes toward new prevention options, anticipated barriers and benefits flags an important issue to consider for a successful introduction of future MPT options. Despite the highlighted limitations, the study provides valuable insights into the experiences and perspectives of those who are proactive in seeking healthcare, offering a positive outlook on the potential effectiveness of targeted interventions for this group. Furthermore, conducting the research in a clinical environment may have influenced participants’ responses, particularly regarding their interest in a multipurpose prevention product. The geographical focus on peri-urban and urban areas might not fully represent attitudes and practices in rural settings. Lastly, the fact that our participants were couples who willingly participated may not be reflective of all couples in these regions. Considering these limitations, further research incorporating a broader range of participants, real-world usage experiences, and a more diverse sample across different settings would be valuable to enhance the understanding of MPT products preferences and address the aforementioned constraints.

## Conclusion

Women of childbearing age continue to suffer the double burden of HIV infection and unplanned pregnancies, highlighting the urgent need for MPT products to address these overlapping health risks. The unique contribution of the study lies in its examination of how perceptions influence preferences for future multipurpose prevention options, specifically focusing on HIV and pregnancy prevention within a relationship context. Couples recognized the benefits of MPTs for HIV and pregnancy prevention but also raised questions about potential side effects. The study highlighted the complex interplay between perceptions, beliefs, and decision-making processes pertinent to future adoption and use of potential MPTs among heterosexual couples. By exploring and understanding these factors, policymakers and healthcare providers can tailor interventions and educational programs to address misconceptions, alleviate concerns, and promote the acceptance and use of MPTs for effective HIV prevention and contraception in the region.

## Data Availability

No datasets were generated or analysed during the current study.
